# The Role of Oxidative Stress and Autophagy in Blue-Light-Induced Damage to the Retinal Pigment Epithelium in Zebrafish In Vitro and In Vivo

**DOI:** 10.3390/ijms22031338

**Published:** 2021-01-29

**Authors:** Kai-Chun Cheng, Yun-Tzu Hsu, Wangta Liu, Huey-Lan Huang, Liang-Yu Chen, Chen-Xi He, Shwu-Jiuan Sheu, Kuo-Jen Chen, Po-Yen Lee, Yi-Hsiung Lin, Chien-Chih Chiu

**Affiliations:** 1 Department of Ophthalmology, Kaohsiung Municipal Siaogang Hospital, Kaohsiung 812, Taiwan; pington64@gmail.com (K.-C.C.); 0870649@kmhk.org.tw (K.-J.C.); 2 Department of Ophthalmology, Kaohsiung Medical University Hospital, Kaohsiung 807, Taiwan; sjiuansheu@gmail.com (S.-J.S.); maco69@gmail.com (P.-Y.L.); 3 Department of Ophthalmology, School of Medicine, College of Medicine, Kaohsiung Medical University, Kaohsiung 807, Taiwan; 4 Department of Biotechnology, Kaohsiung Medical University, Kaohsiung 807, Taiwan; tzu0611@gmail.com (Y.-T.H.); liuwangta@kmu.edu.tw (W.L.); u106500025@kmu.edu.tw (C.-X.H.); 5 Center for Cancer Research, Kaohsiung Medical University, Kaohsiung 807, Taiwan; 6 Department of Medical Research, Kaohsiung Medical University Hospital, Kaohsiung 807, Taiwan; 7 Department of Bioscience Technology, College of Health Science, Chang Jung Christian University, Tainan 711, Taiwan; hhuang@mail.cjcu.edu.tw; 8 Department of Medicine, Kaohsiung Medical University, Kaohsiung 807, Taiwan; verity0418@gmail.com; 9 Division of Cardiology, Department of Internal Medicine, Kaohsiung Medical University Hospital, Kaohsiung 807, Taiwan; 10 Center for Lipid Biosciences, Kaohsiung Medical University Hospital, Kaohsiung 807, Taiwan; 11 Lipid Science and Aging Research Center, Kaohsiung Medical University, Kaohsiung 807, Taiwan; 12 Department of Biological Sciences, National Sun Yat-Sen University, Kaohsiung 804, Taiwan; 13 The Graduate Institute of Medicine, Kaohsiung Medical University, Kaohsiung 807, Taiwan

**Keywords:** retinal pigment epithelium, blue light, DNA damage, apoptosis, reactive oxygen species, autophagy, zebrafish-model, degeneration

## Abstract

Age-related macular degeneration (AMD) is the progressive degeneration of the retinal pigment epithelium (RPE), retina, and choriocapillaris among elderly individuals and is the leading cause of blindness worldwide. Thus, a better understanding of the underlying mechanisms in retinal tissue activated by blue light exposure is important for developing novel treatment and intervention strategies. In this study, blue-light-emitting diodes with a wavelength of 440 nm were applied to RPE cells at a dose of 3.7 ± 0.75 mW/cm^2^ for 24 h. ARPE-19 cells were used to investigate the underlying mechanism induced by blue light exposure. A trypan blue exclusion assay was used for the cell viability determination. Flow cytometry was used for apoptosis rate detection and autophagy analysis. An immunofluorescence microscopy analysis was used to investigate cellular oxidative stress and DNA damage using DCFDA fluorescence staining and an anti-γH2AX antibody. Blue light exposure of zebrafish larvae was established to investigate the effect on retinal tissue development in vivo. To further demonstrate the comprehensive effect of blue light on ARPE-19 cells, next-generation sequencing (NGS) was performed for an ingenuity pathway analysis (IPA) to reveal additional related mechanisms. The results showed that blue light exposure caused a decrease in cell proliferation and an increase in apoptosis in ARPE-19 cells in a time-dependent manner. Oxidative stress increased during the early stage of 2 h of exposure and activated DNA damage in ARPE-19 cells after 8 h. Furthermore, autophagy was activated in response to blue light exposure at 24–48 h. The zebrafish larvae model showed the unfavorable effect of blue light in prohibiting retinal tissue development. The RNA-Seq results confirmed that blue light induced cell death and participated in tissue growth inhibition and maturation. The current study reveals the mechanisms by which blue light induces cell death in a time-dependent manner. Moreover, both the in vivo and NGS data uncovered blue light’s effect on retinal tissue development, suggesting that exposing children to blue light could be relatively dangerous. These results could benefit the development of preventive strategies utilizing herbal medicine-based treatments for eye diseases or degeneration in the future.

## 1. Introduction

Visual defects have a significant negative impact on the quality of life, and this causes an economic burden worldwide. Age-related macular degeneration (AMD) is caused by severe loss of vision in the elderly. AMD is caused by the degeneration of retinal pigment epithelium (RPE) cells and cone photoreceptor cells responsible for light detection in the macula, the central part of the retina. Clinically, AMD can be classified into two major phenotypes, “dry (atrophic) AMD” and “wet (neovascular) AMD”, which involves blood leakage or abnormal angiogenesis. AMD is the most common etiology for which patients attend low-vision clinics.

Lipofuscin, an aggregation of oxidized proteins, was reported to induce apoptosis through proteasome inhibition and pro-apoptotic protein dysregulation [1]. Furthermore, the propensity for RPE cells to be damaged or destroyed by excessive exposure to visible light may be of significance to retinal disorders characterized by enhancing the accumulation of the autofluorescent pigments that constitute lipofuscin, aggregates of oxidized proteins that induce apoptosis through proteasome inhibition and dysregulation of pro-apoptotic proteins [1]. Lipofuscin is a byproduct of RPE cells phagocytosing the lipids constituting photoreceptor cells’ outer segments and accumulating in lysosomes. Lipofuscin is a complex mixture of pigments that are phototoxic to RPE cells [2]. The overaccumulation of autofluorescent lipofuscin could indicate cell aging and increase AMD development risk [3].

RPE cells are highly differentiated unique epithelial cells that interact with adjacent photoreceptors on the apical side and maintain photoreceptor function. RPE cells are located between the layers of photoreceptor cells, providing them with nutrients. If oxidative damage occurs in RPE cells, the breakdown of photoreceptor cells would quickly follow, and visual acuity might become damaged [4]. RPE cells interact with Bruch’s membrane on the basolateral side and the choriocapillaris, which plays an important role in maintaining vision [5]. The damage, metabolic disorder and functional impairment of RPE cells are the key factors in AMD development. Based on past studies, RPE cell impairment could be caused by radiation, oxidative stress, accumulation of lipofuscin, and the immune system [6]. RPE cells are single-layer cells that form parts of the blood barrier and can easily undergo oxidative stress due to their high oxygen consumption. Chronic oxidative stress could induce RPE cell impairment and damage [7]. Among oxidative stresses, photooxidation mediated by lipofuscin notably promotes the development of AMD.

Among visible light, blue light (wavelengths of 400–500 nm) has comparatively higher energy and is more harmful than red light and green light (wavelengths of 500–700 nm) [8]. RPE cells are particularly sensitive to blue light [2], which might exacerbate AMD development [9]. Accumulating evidence indicates that light-induced damage, including blue light and U.V. light, can cause RPE lesions, resulting in or facilitating various macular-associated diseases. Among the spectrum of visible wavelengths (360–830 nm), blue light (400–500 nm) has been reported to facilitate the induction of oxidative stress in the macula [10], and excessive exposure to blue light is associated with the pathophysiology of retinal tissue due to the degeneration of the retina and photoreceptor cells [11]. Short-wavelength light, such as ultraviolet light and blue light, has relatively high energy and may cause severe damage to retinal tissues, especially RPE cells [12,13]. Lipofuscin could promote oxidative damage as a photooxidant when the cells are exposed to blue light. In addition to lipofuscin, there are other potential endogenous substances affected by blue light that may cause phototoxicity to the retina, such as all-trans-retinal and protoporphyrin [14,15].

Oxygenated free radicals with unstable electrons are also known as reactive oxygen species (ROS). Free radicals are atoms, molecules, or ions with an unpaired electron, and they are highly reactive and unstable. ROS can oxidize cells in the body and cause DNA damage or mutation. Common free radicals in cells include the superoxide anion (O_2_^−^), hydrogen peroxide (H_2_O_2_), nitric oxide (NO), and hydroxyl radicals (OH^−•^) [16]. These radicals attack molecules such as DNA, proteins, and lipids, causing damage to cellular tissues [17]. When intracellular ROS is produced in large amounts, and the antioxidant system cannot compete, they will cause oxidative stress. The oxidative stress produced in RPE cells is widely perceived to promote vision-related diseases (e.g., AMD), and oxidative stress is derived from excessive exposure to light, resulting in excessive activation of the visual cycle [18]. According to research, blue light can generate ROS from cytochromes in mitochondria by light induction [19] that inhibits cytochrome oxidase and leads to calcium accumulation and apoptosis of RPE cells [20].

As blue light screens are becoming more common and the dependence on light-emitting diodes (LEDs) is increasing, retinal tissue degeneration in humans caused by blue light has become of increasing concern. Thus, the current study aimed to reveal the underlying mechanism of retinal tissue degeneration regulated by blue light induction to develop a novel treatment strategy or medication for blue-light-induced retinal tissue degeneration.

## 2. Results

### 2.1. Blue Light Exposure Induced ARPE-19 Cell Death by Activating Apoptosis

**Figure 1:**
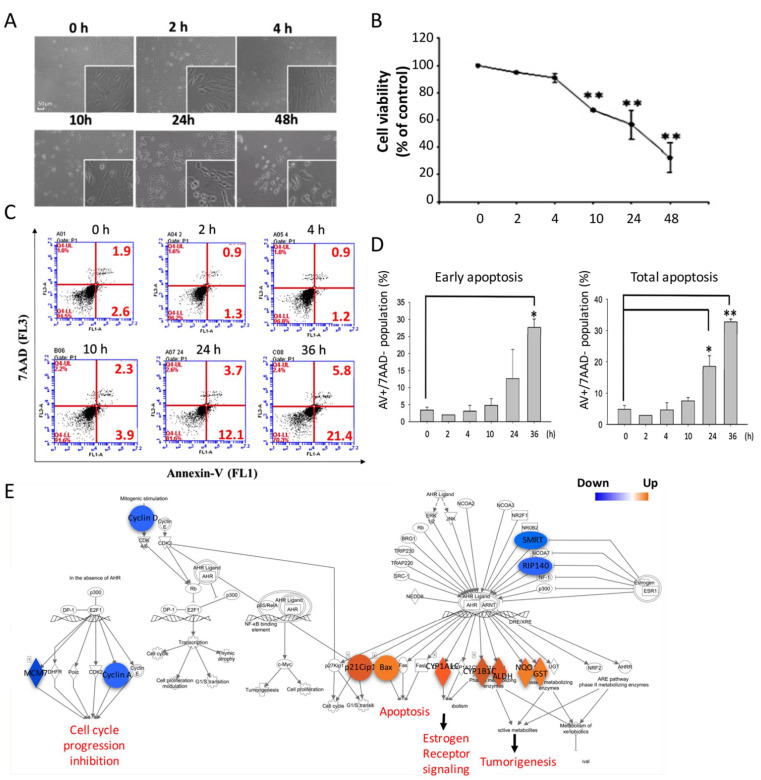
Blue light induced ARPE-19 apoptotic cell death. (**A**) Evaluation of cell viability and death in ARPE-19 cells with time-dependent blue light exposure from 0 h to 48 h (magnification: 40×; enlarged: 200×). (**B**) Blue light induced a decreased ARPE-19 cell viability as determined by an MTS assay (** *p* < 0.01). (**C**) ARPE-19 cell apoptosis induced by blue light exposure for 0 to 36 h. Early and late apoptotic cells were detected using 7AAD and annexin V double staining in a flow cytometry analysis. (**D**) Quantitative results of the flow cytometry analysis of the early apoptosis population (lower right corner) and total apoptosis population (lower right corner) for the statistical analysis (* *p* < 0.05, ** *p* < 0.01). (**E**) NGS (next-generation sequencing) and IPA (ingenuity pathway analysis) were used to determine the potential molecules involved in blue-light-induced apoptotic cell death. Blue: decreased gene expression; orange: increased gene expression.

It has been recognized for decades that blue light is harmful to RPE cells; however, the underlying mechanism remains unclear. In the current study, we aimed to determine the damage caused by blue light in RPE cells and reveal the potential pathways involved. Cell viability alterations were first investigated by using the MTS assay to measure cell survival after exposure to blue light for the indicated durations. As shown in Figure 1A, by observing the cell morphology microscopically, we found that 450 nm blue light exhibited cytotoxicity by decreasing ARPE-19 cell viability in a time-dependent manner. Using the MTS assay, the data showed that blue light induced significant cytotoxicity after 10 h of exposure, and the cell viability was less than 40% after 48 h of blue light exposure (Figure 1B). The results indicate the great potential cytotoxicity of blue light to ARPE-19 cells. 

To investigate the underlying mechanism of damage induced by blue light in ARPE-19 cells, the apoptosis pathway was first determined. By using 7AAD and annexin V double staining, we investigated the early apoptosis activation status. We found that early apoptosis exhibited by the P.S. membrane stain percentage and secondary apoptosis were detected and increased with increasing long-term blue light exposure (Figure 1C). The quantitative data showed that the early apoptosis rate was less than 5% in control ARPE-19 cells and significantly increased to over 26% after 48 h of blue light exposure. The total apoptosis rate also increased significantly from 6% to 33% after 36 h of blue light exposure (Figure 1D). Furthermore, to investigate apoptosis activation by blue light, we performed RNA sequencing to assay apoptosis-related signaling regulation. Through ingenuity pathway analysis^®^ (IPA), the pathways regulated by blue light illumination were revealed. We found that blue light regulated gene activation by increasing gene expression of *p21Cip1*, *Bax*, *CYP1A1*, *ALDH*, *NQO*, and *GST*, and decreasing gene expression of *SMRT*, *RIP140*, *MCM7*, and *Cyclin A* and *D* (Figure 1E). The results suggest that blue light induced significant ARPE-19 cell death during long-term exposure, and apoptosis could be one of the causes of cell death; additionally, cell toxicity might be due to the activation of pro-apoptosis regulators and inhibition of cell cycle checkpoint gene expression.

### 2.2. Oxidative Stress Generation Induced by Blue Light Exposure in ARPE-19 Cells

**Figure 2:**
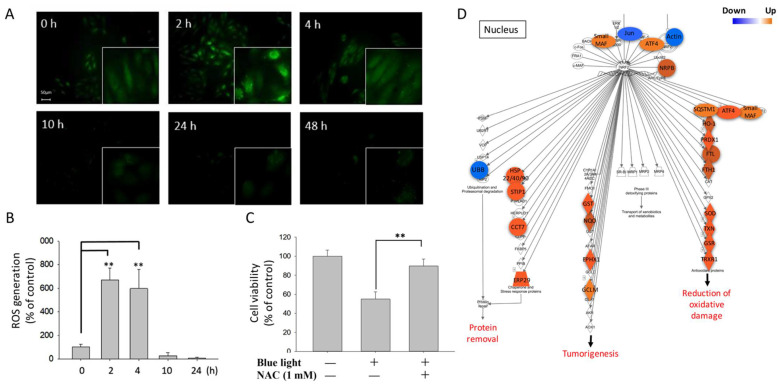
ROS generation in ARPE-19 cells induced by blue light. (**A**) Blue-light-induced ARPE-19 cell ROS (reactive oxygen species) generation was detected by using DCFDA staining and immunofluorescence microscopy. Blue light induction from 0 to 48 h (magnification: 40×; enlarged: 200×). (**B**) Quantitative results of ROS generation induced by blue light within 48 h (** *p* < 0.01). (**C**) MTS assay was used for cell viability determination of blue-light-induced ARPE-19 cell death; the ROS-specific inhibitor NAC (1 mM) was used to inhibit ROS generation (** *p* < 0.01). (**D**) RNA-Seq and IPA of the ROS regulation pathway and molecules involved in ARPE-19 cells affected by blue light exposure. Blue: decreased gene expression; orange: increased gene expression.

To determine the other effects that might be induced by blue light exposure, the generation of ROS was investigated. To measure the ROS levels induced by blue light exposure, DCFDA staining was used. The DCFDA staining morphology of blue-light-exposed ARPE-19 cells was analyzed using immunofluorescence microscopy at the indicated time points, and the data showed that the ROS signals dramatically increased from 2 to 4 h and suddenly dropped to a very low level after 10 h of exposure (Figure 2A). The quantitative data showed that blue light significantly induced an over 6-fold higher ROS signal than that of the control cells after both 2 and 4 h of exposure. The results indicated that blue light induced cellular ROS during early exposure (Figure 2B). To further confirm the role of ROS generation in blue-light-induced cell death, the ROS-specific inhibitor NAC was used for the MTS assay. We found that one mM NAC treatment greatly restored the ARPE-19 cell survival rate by 40% compared to single blue light exposure (Figure 2C). Likewise, we analyzed the underlying mechanism by using RNA-Seq. The results showed that most of the regulators involved in the activation of oxidative stress, such as *small MAF*, *ATF4*, *NRPB*, *SQSTM1*, *HO-1*, *PRDX1*, *FTL*, *FTH1*, *SOD*, *TXN*, *GSR*, *TRXR1*, *GST*, *NQO*, *EPHX1*, *GCLM*, *HSP22/40/90*, *STIP1*, *CCT7*, and *ERP29* gene expression, were upregulated by blue light (Figure 2D). The results suggest that ROS generation could play an important role in the early stage of blue-light-induced cytotoxicity in ARPE-19 cells.

### 2.3. DNA Damage Triggered by Blue Light Stimulation

**Figure 3:**
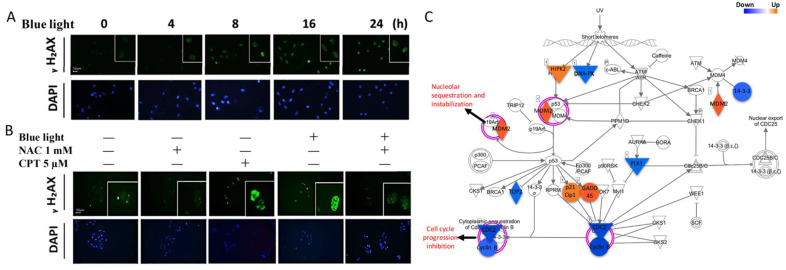
Blue-light-induced ARPE-19 cell DNA damage. (**A**) DNA damage was evaluated by detecting the immunofluorescence intensity of the presented marker, γH2AX. The blue light exposure time was 0 to 24 h (magnification: 40×; enlarged: 200×). (**B**) To confirm the role of ROS generation in DNA damage regulation, the ROS inhibitor NAC (1 mM) was utilized to determine the alteration in DNA damage. CPT (cisplatin; 5 µM) was used in the positive DNA damage control group (magnification: 40×; enlarged: 200×). (**C**) IPA of RNA sequencing showed the molecules potentially involved in ARPE-19 cells regulated by blue light exposure. Blue: decreased gene expression; orange: increased gene expression.

We next investigated DNA damage activation after exposure to blue light. The intensity of the well-known DNA damage marker γH2AX can represent the level of DNA damage induced by blue light. By immunofluorescence staining of γH2AX and microscopic analysis, we found that the expression level of γH2AX significantly increased from 4 to 24 h of blue light exposure. The intense γH2AX immunosignal detected in the nucleus was directly proportional to the blue light exposure time (Figure 3A). To investigate the role of ROS in blue-light-induced DNA damage, the inhibitor NAC was used. Cisplatin (CPT) was used as a positive control that induced cell DNA damage by blocking DNA replication. The results showed low γH2AX expression in ARPE-19 cells under normal conditions, and strong γH2AX expression was found in the nuclei of CPT-treated and blue-light-exposed ARPE-19 cells. However, after 1 mM NAC treatment, γH2AX expression was decreased compared to single blue-light-exposed ARPE-19 cells (Figure 3B). The next-generation sequencing (NGS) data showed that blue light exposure increased gene expression in the cell cycle-related DNA damage response pathway, including HIPK2, MDM2, p21Cip1, and *GADD45*, and decreased *DNA-PK, TOP2*, and *PLK* gene expression (Figure 3C). The results indicate that DNA damage was activated, followed by an increase in oxidative stress and ROS generation.

### 2.4. Cellular Autophagy Activation in ARPE-19 Cells Due to Blue Light Exposure

**Figure 4:**
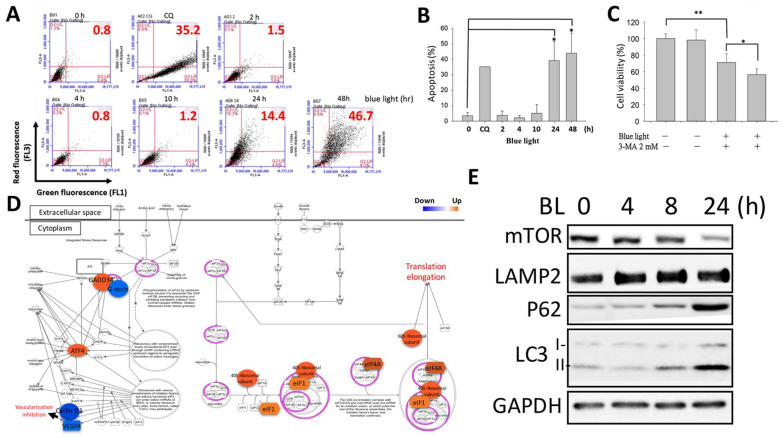
Blue-light-activated ARPE-19 autophagy. (**A**) Flow cytometry was used to determine the autophagic effect induced in ARPE-19 cells by blue light exposure using acridine orange staining. Chloroquine (CQ) was used as a positive control to activate autophagy. (**B**) Quantitative results of blue-light-activated ARPE-19 cell autophagy from 0 to 48 h (* *p* < 0.05). (**C**) To determine the role of the autophagy effect in blue-light-induced ARPE-19 cell death, the autophagy up-stream regulator PI3K inhibitor 3-MA was used to demonstrate cell viability. An MTS (2 mM) assay was used for the cell viability determination (* *p* < 0.05, ** *p* < 0.01). (**D**) RNA-Seq and IPA revealed the underlying mechanism regulated by blue light in ARPE-19 cells. Blue: decreased gene expression; orange: increased gene expression. (**E**) Western blot analysis of the expression of autophagy-related regulator markers, including the initiator inhibitor mTOR, lysosome regulator LAMP2, and autophagosome P62 and LC3 I/II. ARPE-19 cells were exposed to blue light for up to 24 h.

Subsequently, autophagic cell death was determined. Using AO (acridine orange) staining, we detected the autophagy level activated by blue light. Green fluorescence was detected as the original signal inside the cell, and red fluorescence was observed when autophagy occurred in acidic vesicles. Chloroquine (CQ) was used as the positive control to trigger notable autophagy for comparison. As shown in Figure 4A, we found that blue light induced autophagy by increasing the percentage of red fluorescence in ARPE-19 cells in a time-dependent manner. Analysis of the quantitative results showed that autophagy greatly occurred in the 24 and 48 h blue-light-exposed ARPE-19 cells (Figure 4B). 3-Methyladenine (3-MA) was used to inhibit autophagy by blocking PI3K/mTOR activation of autophagosome formation. In the current study, we found that the single treatment of 3-MA did not interfere with cell viability; however, when combined with blue light stimulation, we found that the cytotoxicity was aggravated and increased ARPE-19 cell death by 15% (Figure 4C). RNA-Seq data showed that cell cycle DNA damage-related pathway regulators were modified upon exposure to blue light. Gene expression of ribosomal subunits, including that of the *40S* and *60S* ribosomes, *eIF1, eLF4A, ATF4*, and *GADD34*, was upregulated while *G-actin, cyclin D*, and *VEGFA*, which are involved in cell cycle progression, were downregulated (Figure 4D). The related markers responsible for the different stages of autophagy were determined. The protein expression levels of the lysosome formation marker LAMP2 and the autophagosome maturation markers LC3 I/II and P62 were all upregulated after exposure to blue light up to 24 h. Additionally, the important autophagy repressor mTORC1 was found to be decreased by blue light exposure (Figure 4E). The results suggest that autophagy activated by blue light could be one of the causes of cell death.

### 2.5. The Long-Term Effect of Blue Light Exposure on the Retina of Zebrafish Larvae

**Figure 5:**
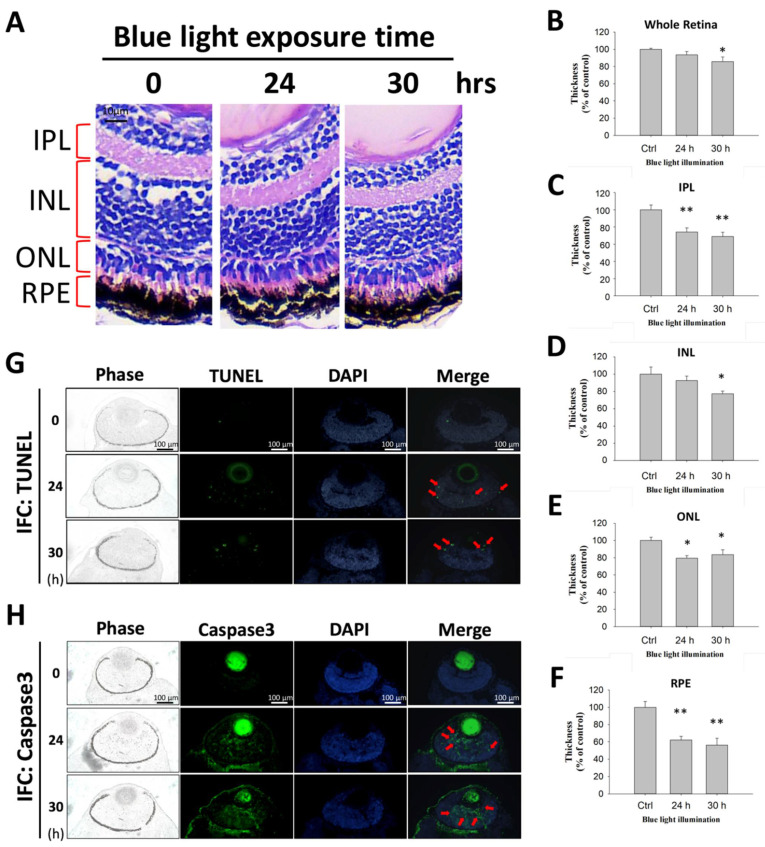
Effect of blue light in the regulation of retinal tissue development in a zebrafish larvae model. (**A**) Retinal tissue sections with HE staining determined the retinal-related layer distribution, including the IPL (inner plexiform layer), INL (inner nuclear layer), ONL (outer nuclear layer), and RPE (retinal pigment epithelium) layers. Blue light exposure times of 24 and 30 h were compared with the 0 h control group. The quantitative thickness results of individual layers, including (**B**) the whole retina, (**C**) IPL, (**D**) INL, (**E**) ONL, and (**F**) RPE layer, were compared to determine the effect of blue light on retinal tissue development (* *p* < 0.05, ** *p* < 0.01). (**G**) TUNEL assay of blue light-exposed zebrafish retinal tissue. Phase: bright-field, Green: TUNEL reaction, Blue: DAPI for nuclear staining. Zebrafish exposed to blue light for 0, 24, and 30 h. (**H**) Retinal tissue of blue-light-exposed zebrafish used for the apoptosis immunofluorescence determination marker caspase-3. Green: Caspase-3 expression.

To determine the harm of long-term exposure of blue light on retinal-related tissue, a zebrafish blue light exposure model was established for retinal histological analysis. As shown in Figure 5A, HE staining of retinal tissue showed that the normal layer distribution of retinal tissue included the inner plexiform layer (IPL), inner nuclear layer (INL), outer nuclear layer (ONL), and RPE layer. However, under blue light exposure for 24 and 30 h, the thickness decreased not only in the whole retina but also in tissues such as the IPL, INL, ONL, and RPE layer. The quantitative results showed that blue light significantly reduced the thickness of all retinal tissue (Figure 5B–F). The harmful effect of blue light on retinal tissue caused retinal cell death and thinned the related tissue layers. To further assay the potential mechanism by which blue light induces cytotoxicity in vivo, zebrafish retinal tissue was used for the TUNEL assay and IFC of apoptosis critical regulator caspase-3 evaluation. Using a TUNEL immunofluorescence assay and microscopy analysis, we found that blue light induced an obvious TUNEL reaction throughout the retinal layers (Figure 5G). Similarly, the IFC caspase-3 protein expression results showed that blue light increased the apoptosis regulator marker’s expression to execute the programmed cell death of retinal cells, including RPE cells (Figure 5H). The results indicate that blue light indeed induced retinal cell death by activating apoptosis signaling in the zebrafish model.

### 2.6. RNA Sequencing to Determine Potential Signal Transduction Involved in Blue-Light-Induced Damage

**Figure 6:**
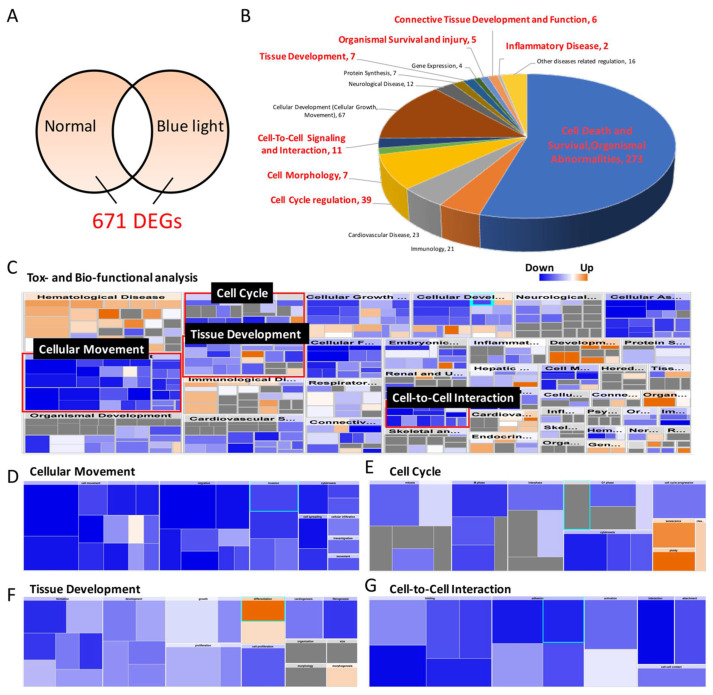
Differential gene expression analysis and the potential mechanisms in ARPE-19 cells regulated by blue light. (**A**) The total number of differentially expressed genes (DEGs) between the control and blue-light-exposed groups. (**B**) Top 15 cellular diseases and functions of the 671 DEGs involved. (**C**) General heatmap of the toxicity and biofunctional analysis generated by IPA and individual catalog heatmaps, including (**D**) cellular movement, (**E**) cell cycle, (**F**) tissue development, and (**G**) cell-to-cell signaling and interaction, were specifically analyzed to determine the regulation pathways affected by blue light. Blue: decreased gene expression; orange: increased gene expression.

To further determine the underlying mechanisms involved in blue-light-induced ARPE-19 cell death, massive gene expression alterations were screened. By using NGS, gene expression in the control ARPE-19 and blue-light-exposed ARPE-19 cells was comprehensively determined. To analyze the differences in gene expression and their roles in blue-light-initiated mechanisms, IPA was used. The pathway enrichment data showed 671 differentially expressed genes after ARPE-19 exposure to blue light (Figure 6A). We found that over 16 cellular processes in ARPE-19 cells were affected by blue light exposure by investigating the related gene alterations and the involved signaling pathways. At least 340 genes (over 241 cancer cell death related, over 67 cellular development related, and 32 cell death survival related) participating in the cell fate decision in ARPE-19 cells were modified by blue light (Figure 6B). The gene expression heatmap showed that blue light exposure inhibited the expression of genes related to normal cell function, behavior, cell–cell interaction, and development by analyzing the effect of blue light on toxic function. Moreover, we found that blue light participated in additional mechanisms, including organismal injury and abnormalities, cancer development, and cell death (Figure 6C). Further analysis showed that the signal transduction related to cellular movement, cell cycle, tissue development, and cell–cell interactions in ARPE-19 cells was comprehensively decreased by blue light cells (Figure 6D–G); the active molecules are shown in Table 1. The RNA-Seq results indicate that blue light’s general inhibitory effect on normal ARPE-19 cell function and its activation of cell-death-related pathways could induce severe harmful effects on all aspects of retinal tissue.

## 3. Discussion

### 3.1. General Discussion

In today’s society, we are inseparable from the computer, communication, and consumer (3C) products. Therefore, we are often exposed to various types of visible light. Blue light in the wavelength range of 400–450 nm is reported to be the most dangerous to the eyes. However, the full mechanism of how blue light induces retinal tissue damage remains undefined. In the current study, we aimed to discover the mechanism involved in blue-light-induced ARPE-19 cell death. Furthermore, gene regulation was extensively screened using NGS to reveal all potential underlying mechanisms affected and regulated by blue light. Our data suggest that blue light reduced retinal ARPE-19 cell survival through at least four phenomena: (1) induced cell apoptosis, (2) ROS generation and increased cellular oxidative stress, (3) DNA damage, and (4) autophagy activation. These four phenomena could be the main reasons contributing to blue-light-induced ARPE-19 cell death.

### 3.2. Blue-Light-Induced Apoptosis

Blue-light-induced retinal cell apoptosis has been reported for decades. Early in 1999 and 2001, Wu et al. and Seko et al. showed evidence regarding rat retinal tissue and cultured RPE cell apoptosis induced by blue light, and both used the same TUNEL assay to evaluate apoptosis and calculate the apoptosis rate in a time-dependent manner. The DNA ladder proved the apoptosis outcome of DNA fragmentation [21,22]. In our case, by using 7AAD and annexin V double staining, we defined the early apoptosis rate and total apoptosis rate by using flow cytometry. However, when we compared the cell survival rate and cell apoptosis rate (early/total), we found that cell viability was significantly decreased after 10 h of blue light exposure, and only slight apoptosis occurred. This phenomenon was interesting, as it indicated that blue-light-induced apoptosis might cause ARPE-19 cell death and participate in other signaling pathways.

### 3.3. Blue-Light-Induced Oxidative Stress

To assess the potential mechanisms of blue-light-induced damage, oxidative stress was investigated. Seko et al. first reported a concept in 2001 that blue light induced rat isolated RPE cell death and damage could be inhibited by using 30 mM NAC [22], and concluded that RPE cells were destroyed through an oxidation-dependent mechanism that eventually led to apoptosis. However, no real data were shown at that time. Immunofluorescence images and quantitative data showed that ROS were generated after only 2 and 4 h of exposure to blue light. These results may explain one of the reasons for decreased cell survival when apoptosis is not fully activated in the early stage of blue light exposure. ROS production could lead to DNA damage and the related inhibition of protein activity that affects the overall cellular process. Lee et al. reported that blue light exposure induced oxidative stress in human corneal epithelial cells and caused a decrease in cell viability [23]. A series of blue light wavelengths were tested to prove the harm of blue light to corneal epithelial cells, and the extract they added effectively inhibited cell death by activating radical scavenging activity and increased the antioxidant enzyme expression of heme oxygenase-1 (HO-1), peroxiredoxin-1 (Prx-1), catalase (CAT), and superoxide dismutase-2 (SOD-2). Thus, blue light might induce the same effect of producing ROS and increasing oxidative stress in RPE cells after short-term exposure.

### 3.4. Blue-Light-Induced DNA Damage

The DNA damage response (DDR) pathway, including the ATM-Chk2 and ATR-Chk1 checkpoints, is activated in oxidative-stress-induced DNA damage to coordinate DNA repair processes, the cell cycle, apoptosis, and cell aging [24]. Ataxia telangiectasia mutated protein (ATM) and ATM- and Rad3-related protein (ATR) are the major regulatory proteins of DDR. Our data clearly demonstrate blue light induced DNA damage through the increased expression of γH2AX. Moreover, NAC treatment strongly suggested that the DDR was mainly through activation of oxidative stress; once a specific inhibitor eliminated the stress, the DNA damage improved. Chen et al. showed evidence in 2019 that blue light could induce DNA double-strand cleavage by upregulating Ku80 expression in blue-light-exposed retinal neurocytes.

### 3.5. Blue-Light-Induced Autophagy

Autophagy is a complex physiological process of degradation and recycling. Nonfunctional or damaged intracellular substances (proteins/lipids/organelles) are engulfed by autophagosomes and degraded in lysosomes. Eventually, they will be reused in the cell metabolic process [25,26]. Several studies have reported that blue light activates autophagy in response to the induced cell damage in related tissues. Early in 2013, Chen et al. found evidence regarding the involvement of autophagy in light-induced Abca4-/-Rdh8-/-mouse autophagy and that the deletion of essential autophagy genes, including Beclin1 and Atg7, further increased cell susceptibility to light-induced retinal tissue damage. The results indicate that autophagy plays a crucial and protective role against light-induced damage [27]. Likewise, Xia et al. specifically highlighted the induction of autophagy by blue light in aged mice. Their data showed that the retinal function in 10-month-old mice changed after five days of blue light exposure, including a delay in *a*-wave and *b*-wave latent periods and a reduction in amplitude. Furthermore, autophagy-related regulators such as PERK, LC3, and Beclin-1 were upregulated in the early stage of blue light exposure and downregulated in the late stage [28]. Our study found that blue light improved autophagy by reducing the expression of the autophagy inhibitor mTOR and increasing autophagy regulators in ARPE-19 cells. The expression of the lysosome formation marker LAMP2 and autophagosome maturation markers LC3 I/II were activated to promote autophagy progression after exposure to blue light for 24–30 h. Blue light is the main component of visible light; thus, our findings in the current study are consistent with the literature that the presence of autophagy might be one of the cellular protection mechanisms against stress-induced cell damage or death in the early stage. P62 is considered the substrate of autophagy, and its increase indicated the abnormal progress of autophagy while exposed to blue light. While the increased lipofuscin was found simultaneously, we believe that the autophagy was blocked after initiation. However, we have proven that the situation could elevate cellular stress and eventually lead to cell death [29]. These findings may explain why the autophagy inhibitor 3-MA combined with blue light exposure could further decrease ARPE-19 cell viability. 3-MA is mainly used to inhibit autophagy progression by prohibiting the formation of autophagosomes and lysosomes; the blocking of autophagosomes could inhibit the autophagy process and an increase in cellular stress. We reported a similar effect of the autophagy-initiating compound C2 ceramide in combination with the autophagy inhibitor CQ. The abnormal increase in cellular stress could eventually turn the protective autophagy effect into a harmful cell death effect [29]. Thus, the phenomena show that the combination of autophagy inhibitors with blue light exposure might cause unexpected, severe damage.

### 3.6. Animal Model

AMD is the most common cause of blindness in elderly individuals worldwide and light-induced AMD could be one of the main reasons for accumulating retinal cell damage and reduced protective effects. Chen et al. used a mouse model to indicate light-induced photoreceptor rod tissue damage in 10-month-old mice, suggesting a promoting effect of light in inducing AMD [27]. In contrast, our zebrafish model shows the effect of blue light on retinal tissue development, which is a different concept than that reported by Chen in 2013. Their data showed a reduction in retinal tissue ONL thickness seven days after light exposure in over 6-month-old or elderly mice. However, by comparing with the control group, we found that multiple layers, including the IPL, INL, ONL, and RPE layer, were significantly affected and exhibited decreased thicknesses after exposure to blue light in the 4-day-old zebrafish larvae. Furthermore, the immunofluorescence assay performed for the TUNEL and caspase-3 investigation demonstrated blue light’s regulatory effect in inducing apoptosis throughout the retinal tissue, revealing the harmful effect of blue light in vivo. This finding was consistent with our in vitro results of blue light’s inhibitory effect on proliferation and the induction of apoptosis in ARPE-19 cells. In the neonatal stages, cell proliferation and differentiation both play important roles in tissue/organ maturation. Thus, the inhibitory effect of blue light impacted cell proliferation and cellular differentiation, leading to more severe sequelae in childhood during retinal development. Related experiments are needed to prove this concept.

### 3.7. NGS Data for Potential Mechanism Prediction

By analyzing RNA sequencing data, we found the molecules involved in blue light regulation in various pathways, including apoptosis, oxidative stress generation, DNA damage, and autophagy, in retinal APRE-19 cells, and a time-dependent analysis showed the chronological order of blue-light-induced effects on cells. Furthermore, the IPA results revealed additional potential mechanisms beyond our expectation: blue light exposure not only induced cell proliferation inhibition but also affected tissue maturation, development, cell–cell interactions, movement, morphology, and inflammation, which have rarely been reported in the past. Combined with our data in zebrafish larvae, blue light likely induced an inhibitory effect on retinal tissue development, causing a reduction in the thickness of multiple layers (whole retina and the IPL, INL, ONL, and RPE layer) of retinal tissue, unlike what was reported for the disorganized photoreceptor outer segment (OS) in Xia’s model focusing on 10-month-old mice. The underlying effects of blue light exposure on retinal tissue growth and development provide valuable information and a scientific basis for future research, and they are worth further validation and discussion.

## 4. Materials and Methods

### 4.1. Culture

The RPE cell line-19 (ARPE-19), a monolayer of polarized epithelial cells located between the sensory retina and choriocapillaris, was differentiated and mitotically inactivated under normal physiological conditions. ARPE-19 cells (BCRC cat. number: 60383), obtained from the Bioresource Collection and Research Center (BCRC, Hsinchu, Taiwan), were grown in Dulbecco’s Modified Eagle’s Medium (DMEM, Invitrogen Corporation, Carlsbad, CA, USA) supplemented with 8% (*v*/*v*) fetal bovine serum, 100 units/mL penicillin and 100 μg/mL streptomycin in an incubator with 5% CO_2_ at 37 °C. The cells were exposed to 0 to 639 J/cm^2^ blue light and cultured for 0 to 48 h. Different blue light exposure dose experiments were performed in triplicate and repeated three times to ensure reproducibility.

### 4.2. Assessment of Cell Viability

The cells (1 × 10^5^) were cultured in 6 cm dishes at 37 °C in a 5% CO_2_ incubator. After 24 h of attachment, the cells were exposed to blue light for 0, 2, 4, 10, 24, 36, or 48 h. The supernatant was removed and washed with 1× PBS; 200 μL of trypsin (Trypsin-EDTA, 2×) (Invitrogen, Carlsbad, CA, USA) was added; and the cells were placed in a 37 °C incubator for 10 min until completely suspended. In total, 1 mL of culture solution was added to the suspended cells in a 15 mL centrifuge tube. The tube was mixed well, 20 μL of the cell solution was transferred to a 1.5 mL centrifuge tube, and then, 20 μL of trypan blue was added and mixed evenly for the cell staining. After the staining, the number of cells was counted under a microscope using a cell counter. The living cells discharged the dye and appeared round and shiny without staining, while the dead cells were stained blue.

### 4.3. Assessment of Cell Viability

Cell viability was assessed using a colorimetric tetrazolium 3-(4,5-dimethylthiazol-2-yl)-5-(3-carboxymethonyphenol)-2-(4-sulfophenyl)-2H-tetrazolium (MTS, Promega, Madison, WI, USA) assay. Briefly, 2.5 × 10^3^ cells were seeded into a 96-well plate, and the cells were treated with different exposure doses of blue light (from 0 to 639 J/cm^2^) for the indicated periods. The final concentrations of MTS and phenazine methyl sulfate (PMS, Sigma-Aldrich, St. Louis, MO, USA) in each well were 80 μg/mL and 7.3 μg/mL, respectively, and the cells were further incubated. Subsequently, the absorbance was measured at 490 nm using a microplate reader (MTX Lab Systems, Inc., Vienna, VA, USA), and the relative cell viability is expressed as the absorbance percentage of the treated relative to that of the control cells.

### 4.4. Determination of Apoptosis by an Annexin V/7AAD Assay

Annexin V (#AVK005, Strong Biotech Corporation, Taipei, Taiwan)/7AAD (#11397, Cayman, Ann Arbor, MI, USA) was used to detect apoptosis. After blue light irradiation, the cells were treated with 10 μg/mL annexin V-fluorescein isothiocyanate and 1 μg/mL 7AAD for 30 min, followed by flow cytometry using an Accuri™ C6 instrument (Becton-Dickinson, Mansfield, MA, USA).

### 4.5. Determination of Intracellular ROS

Cells (1 × 10^5^) were seeded in a 6 cm dish. The cells were treated with blue light irradiation for 0, 2, 4, 10, 24, 36, or 48 h. After the treatment, the cells were incubated with 10 mL of 2,7-dichlorofluorescin diacetate probe (DCFH-DA, Molecular Probes Inc., Eugene, OR, USA) at 37 °C for 20 min and then washed three times with PBS. The fluorescence intensity of DCFH was quantified by flow cytometry (the excitation wavelength was 485 nm and the emission wavelength was 525 nm) using an Accuri™ C6 instrument and its software. The results are presented as the percentage of the control cells.

### 4.6. Assessment of DNA Damage

Cells were exposed to blue light for different durations and the cells were harvested. Then, cell fixation was performed with 4% paraformaldehyde for 10 min and the cells were washed three times with PBS. After 5 min of action with 0.5% Triton, the cells were extracted with 1% BSA by soaking for 15 min, and 500-fold primary antibody γH2AX (Catalog number: sc-101696, Santa Cruz, Dallas, TX, USA) was diluted by adding 1% BSA and was shaken in a refrigerator at 4 °C for one hour. The cells were washed 3 times with 1% BSA for 10 min each time, 1% BSA was added to dilute the secondary antibody 500 times, and the cells were incubated in a refrigerator for 1 h. The secondary antibody was removed, the cells were incubated with 1% BSA and 4′,6-diamidino-2-phenylindole (DAPI) at a concentration of 1 mg/mL, the nuclei were calibrated with 2000-fold diluted DAPI, and the cells were washed five times with 1% BSA. Then, the circular slide was removed from a 24-well plate, 3 μL of oil droplets was added to the slide, and the circular slide was placed vertically on the slide to cover the oil droplets. Finally, an inverted fluorescence microscope (Olympus, Model: IX71, Shinjuku, Tokyo, Japan) was used for imaging.

### 4.7. Acridine Orange Staining for the Assessment of Autophagy

In total, 1 × 10^5^ ARPE19 cells were seeded onto a 60 mm Petri dish in an incubator with 5% CO_2_ at 37 °C. The cells were cultured in 5 mL of medium for 24 h and exposed to blue light for different durations. Trypsin (1 mL) was added to collect the cells, and the cells were centrifuged at 3000 rpm for 10 min at 4 °C. The supernatant was removed, 1 mL of acridine orange (AO) dye was added to the cells for 15 min at 37 °C, and then the cells were centrifuged to remove the supernatant. After washing three times with PBS, the centrifugation step was repeated. The supernatant was removed, 200 μL of PBS was added, and pipetting was carefully and repeatedly performed with an aspirator to suspend the cells. The data were analyzed using a BD Accuri C6 flow cytometer and the accompanying software.

### 4.8. RNA Extraction and Analysis

After the exposure to blue light for 24 h, the total RNA was extracted by TRIzol^®^ reagent (Invitrogen, USA) according to the manufacturer’s instructions. The purified RNA was quantified by a nanodrop 2000c spectrophotometer (Thermo Scientific, Waltham, MA, USA) to obtain the optical density (OD) at 260 nm, and a Qubit 2.0 fluorometer (Life Technologies, Carlsbad, CA, USA) was used with an Agilent 2100 Bioanalyzer system (Agilent Technologies, Santa Clara, CA, USA) to assess the RNA integrity.

### 4.9. Library Preparation and Sequencing

The RNA library construction and sequencing were performed by Tools Biotech (BIOTOOLS, Taipei, Taiwan). According to the manufacturer’s instructions, the total RNA was used for the library construction using a TruSeqtm RNA LT sample preparation kit (Illumina Inc., San Diego, CA, USA). Magnetic beads labeled oligo (dt) were used to collect mRNA from the ARPE-19 cells in the different treatment groups. The mRNA was then randomly fragmented in fragmentation buffer, and cDNA synthesis was performed using random hexamers and reverse transcriptase. After the first strand synthesis, a custom second strand synthesis buffer (Illumina), dNTPs, RNase H, and *E. coli* polymerase I were added to form a second strand by nick translation. After purification, repair of the terminal, A-tailing, sequence adaptor ligation, size selection, and PCR enrichment, the final cDNA library was complete. Subsequently, the library concentration was first quantified using a Qubit^®^ dsDNA H.S. assay kit (Life Technologies, Carlsbad, CA, USA), followed by dilution to 1 ng/μL using an Agilent 2100 Bioanalyzer system (Agilent Technologies, Santa Clara, CA, USA). The cDNA size in the library adapters was determined at both ends and quantified to relatively high accuracy (library activity > 2 nm) by quantitative PCR (Q-PCR). The RNA libraries were sequenced on an Illumina HiSeq 2500 (Illumina, Inc., California, CA, USA) instrument for 300 cycles. The sequencing data were processed using Illumina software HiSeq X system (HD.3.4.0/RTA 2.7.7).

### 4.10. Biological Pathway Analysis

First, the original fastq readings were used to verify the determined sequence quality. The adapter sequences were predeleted, the low-mass end was trimmed, and Trimmomatic software was used to filter the low-quality readings with Q33. Then, the low-quality data from tophat2 software were used to generate RNA. For the sequence data analysis, the high-quality reading alignment was compared to the human reference genome (grch38.p7). The gene counts were calculated by featureCounts software, and RLE/TMM/FPKM normalized the performance. The differentially expressed genes were screened according to a *q*-value < 0.05 as a screening threshold. The differential gene results were screened, and downstream systematic bioassays were performed using the Kyoto Encyclopedia of Genes and Genomes (KEGG) pathway database (http://www.genome.ad.jp/kegg/kegg1.Html).

### 4.11. Statistical Analysis

All experiments were conducted in triplicate and the data are represented as the mean ± S.D. The mapping and statistical analysis were performed using Sigma Plot 12.0 (SPSS Inc., Chicago, IL, USA). The data were subjected to an analysis of variance (ANOVA) and Duncan’s multiple range tests. *p* < 0.05 was considered significant.

## 5. Conclusions

**Figure 7:**
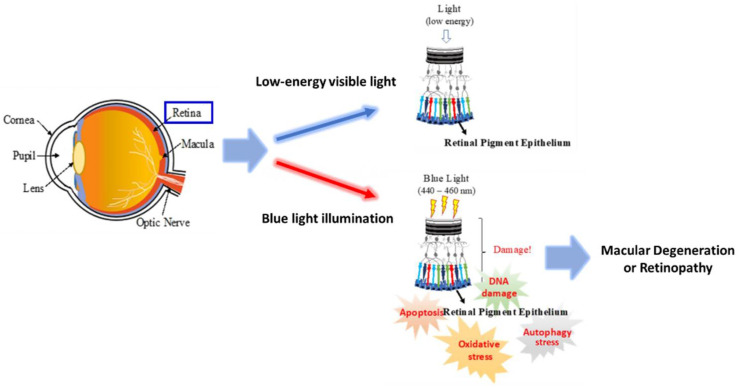
Schematic illustration of blue-light-induced pathogenesis of retinal tissue. Our results suggest that blue light induced a sequential effect in RPE cells, including (1) ROS generation, (2) DNA damage, (3) autophagic stress, and (4) apoptosis, eventually resulting in macular degeneration or retinopathy. The blue arrow indicates low energy visible light which is non-harmful to RPE and the red arrow indicates the illumination of high-energy blue light.

In conclusion, our results suggest that blue light’s harmful effect is based on the induction of retinal ARPE-19 cell death through a sequential mechanism of cell proliferation inhibition and the induction of DNA damage and oxidative stress (Figure 7). Although the protective mechanism autophagy simultaneously occurs with cell proliferation inhibition and oxidative stress, it seems insufficient for preventing the accumulation of damage. More likely, the tracking process of autophagy could be one of the reasons that brought the RPE cell to autophagic cell death due to the pressure of cellular stress. The zebrafish larvae model suggests that blue light’s harmful effect includes reducing retinal tissue layer thickness and retinal degeneration in 4-day-old zebrafish in which retinal tissue is still developing. The results of RNA-Seq provided many potential mechanisms that participated in the effect of blue light on ARPE-19 cells, indicating impacts on not only cell survival and cell cycle regulation but also the regulation of cellular/tissue development, cell–cell interactions, protein/gene expression, and inflammation. The current study revealed novel evidence regarding blue light damage to retinal cells, as shown in the graphic abstract, and a scientific basis for developing a new therapeutic strategy against blue-light-induced macular degeneration in modern society.

## Figures and Tables

**Table 1:** Molecules involved in blue-light-induced ARPE-19 cellular function.

Categories	Predicted Activation State	*p*-Value	Activation z-Score	Involved Molecules
**Cellular** **Movement**	Decreased	9.36 × 10^−47^	−4.498	ACTB, ACTN1, ACTN4, ACTR2, ADAM19, ADAM9, ADAMTS5, AHNAK, AKAP12, AKT1S1, ALCAM, ALDH1A3, ALDOA, ANGPTL2, ANXA2, ANXA3, ARHGAP11A, ARL4C, ARPC2, ASPH, ASPM, AXL, BAX, BTG2, CA9, CALD1, CALR, CAP1, CAST, CCBE1, CCN1, CCN2, CCNA2, CCND1, CD274, CD44, CD63, CDH11, CDH4, CDK1, CDKN1A, CENPF, CFL1, CLIC4, CNN2, COL11A1, COL4A1,COL4A2, CRMP1, CRY1, CSF1, CTSL, CYP1A1, CYP1B1, DAG1, DCBLD2, DDRGK1, DEK, DIAPH1, DIO2, DKK1, DNAJA1, DNAJB4, DSP, DUSP1, ECT2, EDN1, EFNB2, EGFR, EGLN1, EMP1, ENAH, ENO1, ENPP2, EPPK1, ERRFI1, ETS1, EZR, F2R, FASN, FAT1, FAT3, FBN1, FERMT2, FGF1, FGF5, FKBP4, FLNA, FLNB, FLNC, FLRT2, FN1, FOSL2, FOXF2, FOXQ1, FSTL1, GADD45A, GAPDH, GAS5, GDF5, GIPC1, GNAI2, GNG12, GPC1, GPI, GTSE1, HIF1A, HMGA2, HMGB1, HMGB2, HMOX1, HSP90AA1, HSPA8, HSPB1, HSPD1, HSPG2, ID1, ID3, IGF2R, IGFBP3, IGFBP5, IGFBP7, IL7R, INHBA, INPPL1, IRS2, ITGA3, ITGA4, ITGA5, ITGAV, ITGB1, ITGB3, JUN, JUNB, KDM3A, KIF14, KIF20B, KIFC1, KLF2, KLF6, KPNA2, KRT18, KRT8, LCP1, LDHA, LIMA1, LMNB1,LMNB2, LMO4,LMO7, LOX, LOXL2, LRP1, LTBP2, LTBP3, LTBR, MAP1B, MAP2, MAP4, MBOAT7, MDM2, MIR100HG, MSN, MYADM, MYH10,MYH9, MYL12A, NAV1, NDST1, NEDD9, NEU1, NGF, NINJ1, NKX3-1, NOG, NPPB, NQO1, NR3C2, NREP, NRP1, OSGIN1, P4HB, PALLD, PCOLCE2, PDGFB, PDK1, PHLPP1, PLXNB2, PODXL, PPIF, PPP1R15A, PRDX1, PTEN, PTPRB, PTPRF, PTPRJ, RACGAP1, RELN, RPL13A, RPS19, RUNX2, SEMA3B, SEMA7A, SERPINB2, SERPINE1, SERPINE2, SGK1, SLC16A3, SLC3A2, SLC48A1, SLC7A11, SLC7A5, SLIT3, SOD1, SORT1, SOX9, SPARC, SPHK1, SQSTM1, TAGLN2, TFPI, TGFB2, TGFBR1, THBS1, TIMP3, TKT, TLN1, TMEM30A, TMPO, TMSB10/TMSB4X, TNFRSF10B, TNIK, TNS1, TPI1, TPM1, TPM2, TPM3, TPT1, TSPAN14, TUBA1C, TUBB, TXN, TXNRD1, UCHL1, VASP, VCL, VEGFA, VIM, VSIR, WBP2, WWC1, YBX1, ZYX
**Cell Cycle**	Decreased	1.49 × 10^−23^	−2.301	AKAP12, ANLN, BIRC5, BUB1B, CAP1, CCNB1, CDC20, CDK1, CDKN1A, CENPE, CEP55, CFL1, CIT, CKAP2, CSF1, DIAPH3, ECT2, FLNA, GADD45A, GIPC1, GNAI2, GPC1, HIPK2, HSPB1, INCENP, ITGB1, KIF14, KIF20A, KIF20B, KIF23, KIFC1, MCM7, MDM2, MYH10, NDC80, NEK6, NEK7, NUSAP1, PLK1, PRC1, RACGAP1, SPART, SPDL1, TACC3, TM4SF1, TOP2A, UBE2S, YBX1
**Tissue** **Development**	Decreased	7.58 × 10^−16^	−1.174	ANXA2, AXL, BMPER, CA9, CALR, CCN1, CCND1, CD44, CDKN1A, CLIC4, COL4A2, CSF1, DAG1, DKK1, ECT2, EDN1, EFNB2, EGFR, F2R, FAT1, FGF1, FLNB, FN1, FOSL2, FRAS1, GPC1, HEG1, HIF1A, HIPK2, HMGB1, HMGB2, HMOX1, HSPG2, ID1, ID3, IGFBP3, ITGA4, ITGB1, ITGB3, JUN, KRT18, KRT8, LOXL2, LTBR, MYDGF, NGF, NKX3-1, NRP1, ODC1, PALLD, PDGFB, PLOD3, PTEN, PTPRJ, RUNX2, SERPINE1, SOX9, SPARC, TGFBR1, THBS1, TIMP3, VCL, VEGFA
**Cell-Cell Signaling and Interaction**	Decreased	7.45 × 10^-15^	−1.439	ADAM9, AHNAK, ANXA2, AXL, BAMBI, BAX, BHLHE40, BMPER, BNIP3, CALR, CCN1, CCN2, CCND1, CD274, CD44, CDKN1A, CFL1, CRY1, CSF1, CTSL, DKK1, DUSP1, EDN1, EFNB2, EGFR, ETS1, F2R, FBN1, FERMT2, FGF1, FLNA, FN1, GADD45A, GAS5, GNAI2, HMGB1, HSPB8, HSPD1, HSPG2, HSPH1, ID1, ID3, ITGA3, ITGA4, ITGA5, ITGAV, ITGB1, ITGB3, KLF2, KRT18, KRT8, LOX, LRP1, LTBR, MGST1, MT-CO1, MT-ND2, MT-ND3, MT-ND4, MT-ND5, NBR1, NCOR2, NGF, NPPB, ODC1, PDGFB, PFKFB3, PHLPP1, PLXNB2, PRDX1, PRKDC, PTEN, PTPRJ, RAB3B, SERPINE2, SOD1, TAB3, TACC3, TGFB2, TGFBR1, THBS1, TLN1, TNFRSF10B, TOP2A, TPT1, VASP, VEGFA, VIM, VSIR

## Data Availability

Not applicable.
